# Candidemia in critically ill immunocompromised patients: report of a retrospective multicenter cohort study

**DOI:** 10.1186/s13613-019-0539-2

**Published:** 2019-06-03

**Authors:** Etienne Ghrenassia, Djamel Mokart, Julien Mayaux, Alexandre Demoule, Imène Rezine, Lionel Kerhuel, Laure Calvet, Audrey De Jong, Elie Azoulay, Michael Darmon

**Affiliations:** 1 Medical ICU, Saint Louis Hospital, Paris, France; 2 Paris Diderot University, Paris, France; 3 Sorbonne University, Paris, France; 4 ICU, Paoli-Calmettes Institute, Marseille, France; 5 Medical ICU, Pitié-Salpétrière Hospital, Paris, France; 6 Département d’anesthésie-réanimation Saint-Eloi, PhyMedExp, INSERM, CNRS, CHU de Montpellier, Université de Montpellier, 80, avenue Augustin-Fliche, 34295 Montpellier Cedex, France; 7 ECSTRA Team, Biostatistics and Clinical Epidemiology, UMR 1153 (Center of Epidemiology and Biostatistic Sorbonne Paris Cité, CRESS), INSERM, Paris, France; 8 Public Health Department, Robert Debré University Hospital, AP-HP, Paris, France

**Keywords:** *Candida*, Hematological malignancy, Tumor, Solid, Immune defect, Intensive care unit, Shock

## Abstract

**Background:**

Immunocompromised critically ill patients constitute a population with the high risk of candidemia. This retrospective study aimed to assess the outcome of immunocompromised critically ill patients with candidemia. Secondary objectives were to describe clinical phenotypes of these patients, *Candida* ecology, and factors associated with mortality.

**Results:**

Overall, 121 patients were included in this study. Median delay from candidemia to first antifungal therapy was 3 days, in line with the observed delay of blood culture positivity. *Candia albicans* was the main *Candida* specie identified (54%), and susceptibility of *Candida* to fluconazole and echinocandins was of, respectively, 70% and 92%. Hospital mortality was of 60%. After adjustment for confounders, severity as assessed by the need for vasopressors (HR 1.8, CI95% 1.1–3.1), need for mechanical ventilation (HR 2.0, CI95% 1.1–3.8) and allogenic stem cell transplantation (HR 2.5, CI95% 1.1–6.0) were independently associated with poor outcome. *Candida* specie, susceptibility and treatment strategies were not associated with outcome.

**Conclusions:**

Candidemia in immunocompromised critically ill patients is associated with a grim outcome. Despite the high prevalence of *Candida* non-*albicans* species, neither *C.* species nor its susceptibility was associated with outcome. Conversely, severity and preexisting allogeneic stem cell transplantation were independently associated with poor outcome. Despite antifungal prophylaxis and use of preemptive antifungal therapy in neutropenic patients, antifungal therapy was initiated three days after symptoms onset suggesting needs for specific strategies aiming to reduce this delay.

**Electronic supplementary material:**

The online version of this article (10.1186/s13613-019-0539-2) contains supplementary material, which is available to authorized users.

## Background

Candidemia represents 10% of nosocomial infections in hospitalized patients and is associated with mortality described to be as high as 40% [1–4]. Underlying immune defect, solid or hematological malignancy, may predispose to candidemia which develops during the clinical course of these conditions in 1.8% of cases [5].

Despite being widely studied, several areas of uncertainty remain. First, diagnosis of candidemia is often delayed as consequences of time required to obtain blood cultures positivity. Although several studies suggested benefit of early initiation of antifungal therapy on patients survival [6–8], evidence supporting benefits of preemptive treatment in high-risk critically ill patients is lacking [9, 10]. Antifungal resistance among documented *Candida species* is growing, and resistance to fluconazole and echinocandins has been described in 20% and 6.5% of Candida, respectively [4]. Last, patients’ severity and comorbidities, such as immunosuppression, are known risk factor for candidemia [11] which may participate in the observed grim prognosis of candidemia.

Half of the patients with candidemia are critically ill [1, 4], mortality in patients with hemodynamic instability reaching 70% [7]. Underlying malignancy, either hematological or solid tumors, is frequently associated with candidemia, half of the patients with candidemia having underlying malignancy [4]. These patients may have specific risk factors for candidemia such as catheter or neutropenia, risk factors for fluconazole-resistant species such as prophylaxis, or specific acute condition such as typhlitis [12]. Nonetheless, immunocompromised critically ill patients with candidemia have been poorly described except in specific subgroups such as HIV infected patients [12] or organ transplant recipients [13].

The primary objective of this study was to assess outcome of immunocompromised critically ill patients with candidemia. Our secondary objectives were to describe clinical features of these patients and fungal ecology and to identify prognostic factors in this setting.

## Methods

### Study population

This study is a retrospective multicenter observational study, in three ICUs located in two university hospitals (Saint-Louis and Pitié-Salpétrière Hospitals, Paris, France) and a comprehensive cancer center (Paoli-Calmettes Institute, Marseille, France). Adult patients (age > 18 years) admitted in participating centers between January 2002 and December 2017 and who developed a candidemia 72 h before ICU admission or during ICU stay were included. Patients for whom HIV infection or solid organ transplantation was the only identified immune defect were excluded. Patients with invasive candidiasis without candidemia were also excluded.

This study was approved by the French Intensive Care Society ethics committee (CE-SRLF-18-06). Need for informed consent was waived as regard to the study observational design and in accordance with the French law. Patients alive at time of the analysis were, however, informed, and none refused to participate. This study was conducted in accordance with the principles of the Declaration of Helsinki.

### Protocol and definitions

**Fig. 1:**
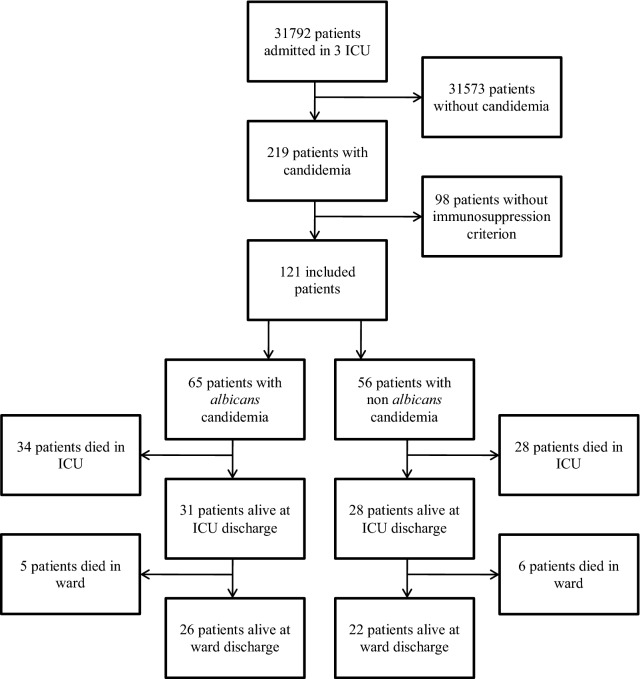
Flow chart

Patients were identified retrospectively through ICUs diagnostic files (Fig. 1).

Considering immune defect, this study intends to study patients with hematological malignancy, solid tumors or immunosuppressive drug for underlying autoimmune disease or vasculitis. Patients with solid organ transplantation or HIV as sole source of immune defect were excluded.

*Candida* colonization was defined as identification of *Candida* in at least one site including skin, urines, lung, mouth or on rectal swab.

Onset of candidemia was defined as the delay between blood sample leading to candidemia identification and antifungal therapy initiation.

Preemptive antifungal therapy was defined as antifungal therapy initiated the day of blood sampling.

A dedicated form was used to report:

1. Demographic data such as age, sex, type of immunosuppression; identified risk factors for candidemia as antifungal prophylaxis, recent abdominal surgery, recent renal replacement therapy, parenteral nutrition, presence of arterial or central venous catheter [14]; intensive care unit features as SOFA at day 1, need for vasopressors and for mechanical ventilation and renal replacement therapy;
2. Candidemia features as day of first and last positive blood culture, delay of positivity, *Candida* specie, fluconazole and echinocandins susceptibility based on EUCAST 2017 breakpoints [15], first and last line treatment, time to treatment introduction, time to catheter ablation, secondary localizations and delay between onset of candidemia and treatment initiation;
3. Outcomes as intensive care unit mortality, hospitalization mortality, mortality on the last follow-up.

### Statistical analysis

We first performed a descriptive analysis in order to identify global characteristics of included patients. Data are reported as median and interquartile range or number (%).

Factors associated with hospital mortality were identified using univariate analysis. Chi-square test or Fisher’s exact test, as appropriate, was used for categorical variables. Mann–Whitney or Wilcoxon rank-sum tests, as appropriate, were used for continuous variables. A conditional Cox model was used to identify factors independently associated with hospital mortality. Variables yielding *p* values less than 0.2 in the univariate analysis or considered clinically relevant were entered in a backward stepwise model. Critical removal *p* value was of 0.1. Only 1 variable for 7 events was included in the model in order to avoid any risk of overfitting. Proportional hazards assumption was confirmed by checking scaled Schönfeld residuals against time and correlations between covariates were searched for. Last, we planned previous the analysis to force in the final model, should these variables not be selected *Candida* species (*C. albicans* vs. *C. non*-*albicans*) and to force treatment strategy (echinocandin as first-line therapy). As a post hoc analysis, ICU admission year (per quartile of ICU admission year) was forced in the final model.

Survival was plotted using Kaplan–Meier curves, and differences were assessed using log-rank test.

Last, in a way to assess the influence of candidemia on outcome, patients of this study were compared to patients in the EFRAIM dataset [16], after exclusion of patients with candidemia in this later and after exclusion having as sole immune defect solid organ transplant. Raw mortality was compared; then matching was performed on relevant variables using propensity score matching, according to closest neighbor methods and aiming to a 1:1 case–control ratio. Patients with and without candidemia were compared before and after matching according to standardized mean difference. Unadjusted mortality before matching, after matching and after matching and after adjustment for SOFA score was performed using Kaplan–Meier curves, log-rank test and Cox model.

All tests were two-sided, and *p* values less than 0.05 were considered statistically significant.

All statistical tests were performed using R software (https://www.r-project.org/); ‘matchIt’ and ‘survival’ package.

## Results

### Patients’ characteristics

Overall, among the 31,792 patients admitted in the participating ICUs during the study period, 219 developed a candidemia (0.7%). Overall, 121 had an underlying immune defect as defined by our protocol and were included (Fig. 1).

Main patients’ characteristics are reported in Table 1 and Additional file 1: Table S1.

**Table 1:** Hospital mortality risk factors (number (%) or median (IQR))

Clinical features	Total *N* = 121	Dead *N* = 73	Alive *N* = 48	*p* value
Female gender	47 (39%)	24 (33%)	23 (48%)	0.13
Age	60 (49–66)	60 (49–65)	61 (52–68)	0.3
Median year of ICU admission (IQR)	2013 (2010–2015)	2013 (2010–2016)	2013 (2010–2015)	0.86
Underlying immunosuppression				
Solid tumors	36 (30%)	19 (26%)	17 (35%)	0.31
Hematological malignancy	81 (67%)	52 (71%)	29 (60%)	0.24
*Allogenic SCT*	8 (7%)	7 (10%)	1 (2%)	0.14
*Acute myeloid leukemia*	17 (14%)	10 (14%)	7 (15%)	1
*Acute lymphoid leukemia*	9 (7%)	7 (10%)	2 (4%)	0.32
*Lymphoma*	45 (37%)	29 (40%)	16 (33%)	0.56
*Myelodysplasia*	6 (5%)	4 (5.5%)	2 (4%)	1
Autoimmune disease	13 (11%)	7 (10%)	6 (12.5%)	0.77
HIV infection	19 (16%)	11 (15%)	8 (17%)	0.80
Neutropenia	58 (49%)	37 (51%)	21 (46%)	0.71
ICU features				
SOFA score	10 (6–15)	12 (8–16)	8 (5–14)	0.011
Surgical patient	25 (21%)	15 (20%)	10 (21%)	1
Renal replacement therapy	71 (61%)	50 (68.5%)	22 (46%)	0.005
Invasive mechanical ventilation	91 (75%)	61 (85%)	31 (65%)	0.015
Vasopressors	66 (54.5%)	45 (62%)	21 (44%)	0.06
Candidemia features				
Antifungal prophylaxis	20 (17%)	16 (22%)	4 (8%)	0.08
ICU acquired candidemia	70 (58%)	44 (63%)	26 (54%)	0.5
*Candida albicans*	65 (54%)	39 (53%)	26 (54%)	1
*Candida glabrata*	23 (19%)	13 (18%)	10 (21%)	0.81
*Candida tropicalis*	13 (11%)	9 (12%)	4 (8%)	0.56
*Candida krusei*	9 (7%)	7 (10%)	2 (4%)	0.32
*Candida parapsilosis*	9 (7%)	5 (7%)	4 (8%)	0.74
Fluconazole susceptibility	61 (70%)	35 (71%)	27 (69%)	1
Echinocandins susceptibility	73 (92%)	39 (89%)	34 (97%)	0.22
Adequacy of first AF therapy	78 (90%)	43 (86%)	36 (94%)	0.29
CVC removed	103 (97%)	61 (98%)	42 (95.5%)	0.57
Days to first AF therapy (IQR)	3 (1–3)	3 (1–3)	3 (1–3)	0.45
Preemptive antifungal therapy	27 (22%)	16 (22%)	11 (23%)	1.00

Adequacy is based on antifungal susceptibility of involved *Candida* based on MIC

*ICU* intensive care unit, *IQR* interquartile range, *SCT* stem cell transplantation, *AF* antifungal therapy, *CVC* central venous catheter

Median age was 60 years (IQR 49–66), and 74 (61%) were of male gender. Two-third of the patients had hematological malignancy, lymphoma (37%) and acute leukemia (21%) being main underlying diseases. Respectively, 10% and 7% of included patients were autologous or allogenic stem cell transplant recipients. Among solid tumors, breast (25%), lung (11%) and gynecological (11%) cancers were the most prominent.

Most of the patients had one or several risk factors for candidemia including *Candida* colonization (81%), presence of central venous catheter (94%), need for renal replacement therapy (38%), parenteral nutrition (30%) or recent abdominal surgery (12%). Similarly, several immune defects were frequently associated including HIV infection (16%), known hypogammaglobulinemia (10%) and neutropenia (49%). Interaction between the main risk factors is reported in Additional file 1: Figure S1.

At ICU admission, severity as assessed by SOFA score was 10 (6–15). The vast majority of patients had a medical condition, and 25 (21%) were admitted for a surgical emergency. Half of patients (54.5%) required vasopressors at ICU admission.

During ICU stay, 91 (75%) patients required invasive mechanical ventilation, 71 (61%) required renal replacement therapy, and 91 (75%) required vasopressors.

### Candidemia characteristics

Half of the patients (42%) were admitted in ICU after onset of the candidemia, the remaining patients having ICU acquired candidemia (Additional file 1: Table S2). Proportion of patients with breakthrough candidemia (under antifungal prophylaxis) was 16.5%. Main characteristics of candidemia are reported in Additional file 1: Table S1. *Candida albicans* (54%) was the predominant specie, followed by *C. glabrata* (19%), *C. tropicalis* (11%), *C. parapsilosis* (7%) and *C. krusei* (7%). One-third of identified *Candida* was resistant to fluconazole (30%) and 8% to echinocandins.

The median delay to blood culture positivity was 3 days (range 1–6). Enterocolitis was the most frequent infection site (55%). Among the 99 cultured catheters, 18 (18%) were colonized or infected. Secondary localizations included endophthalmitis (10%), cutaneous lesions and thrombosis (8% each). Despite being searched for, no patient developed osteo-articular infection or endocarditis. Chronic disseminated candidiasis with hepatosplenic lesions was described in two patients (2%).

First-line antifungal therapy was adapted to documented specie in the vast majority of the patients (90%), and central venous catheter was nearly systematically removed (97%) within 3 days (IQR 2–4). Median delay from onset of candidemia to first antifungal therapy was 3 days (IQR 1–3).

### Outcome and factors associated with hospital mortality

**Fig. 2:**
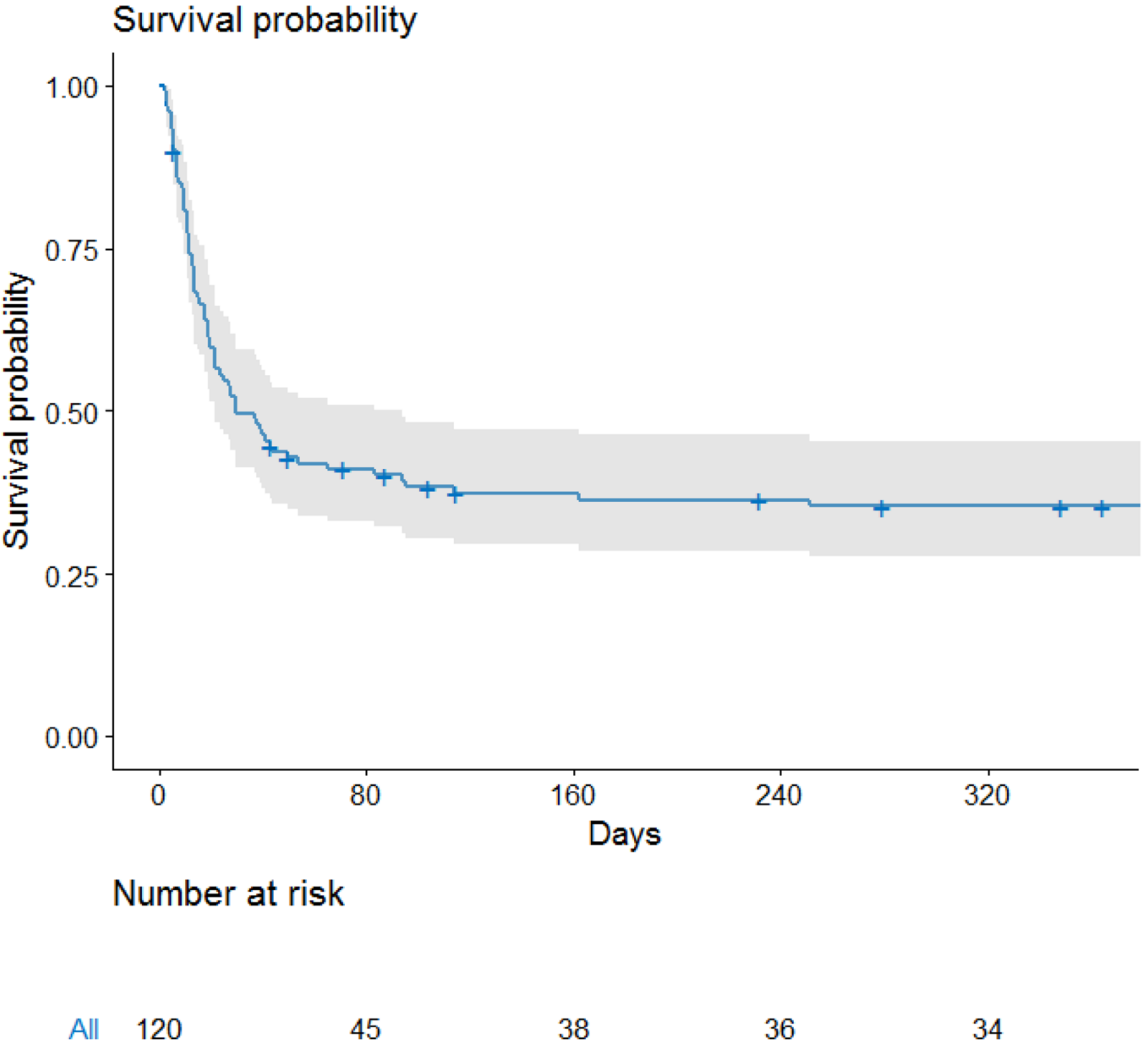
Kaplan–Meier survival curve

ICU mortality was of 52%, and hospital mortality was of 60% (Fig. 2). Before adjustment, patients’ severity as assessed by organ support or severity score was the main variables associated with hospital mortality (Table 1).

**Fig. 3:**
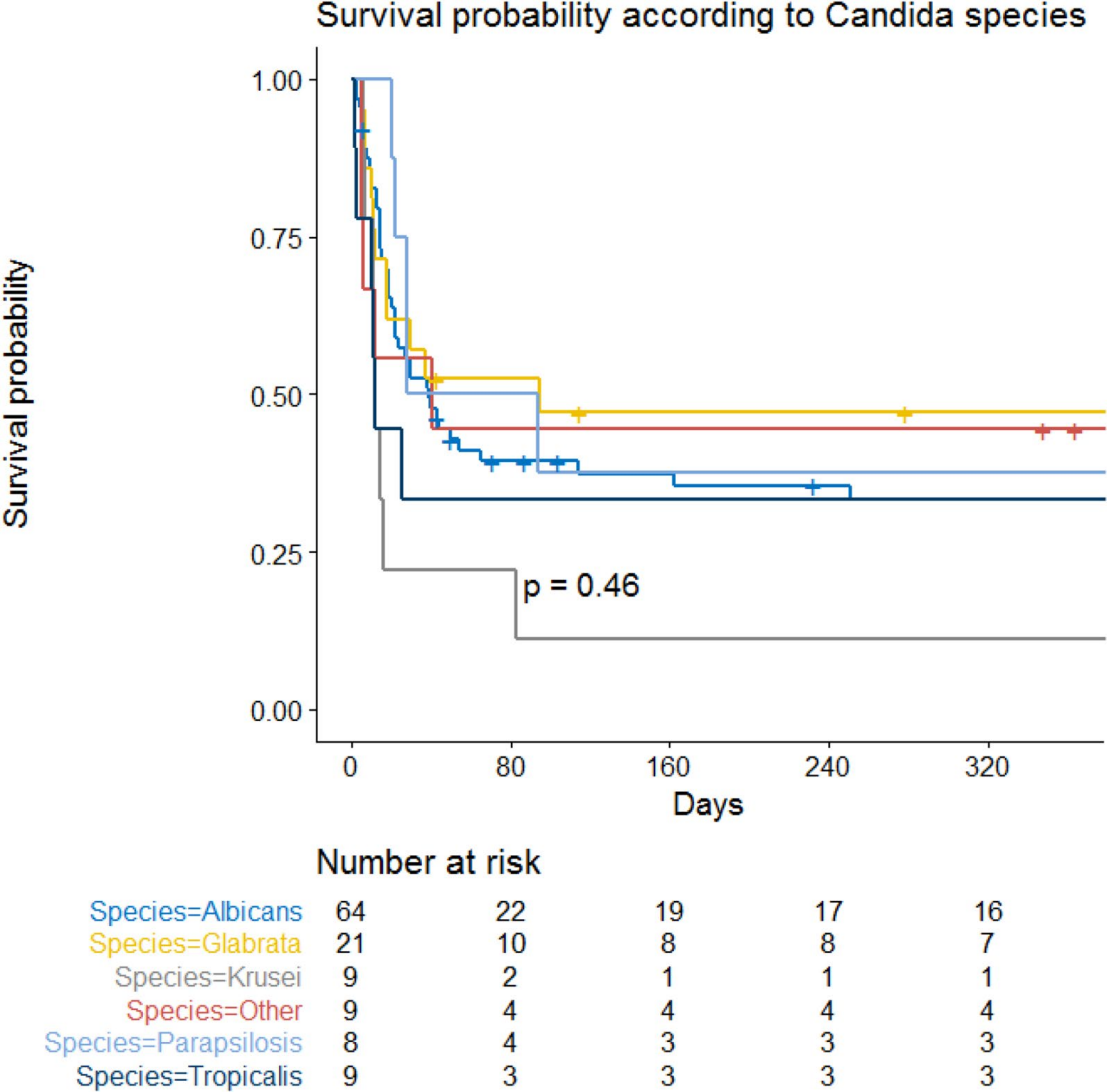
Kaplan–Meier survival curve. Survival probability according to *Candida* species

**Fig. 4:**
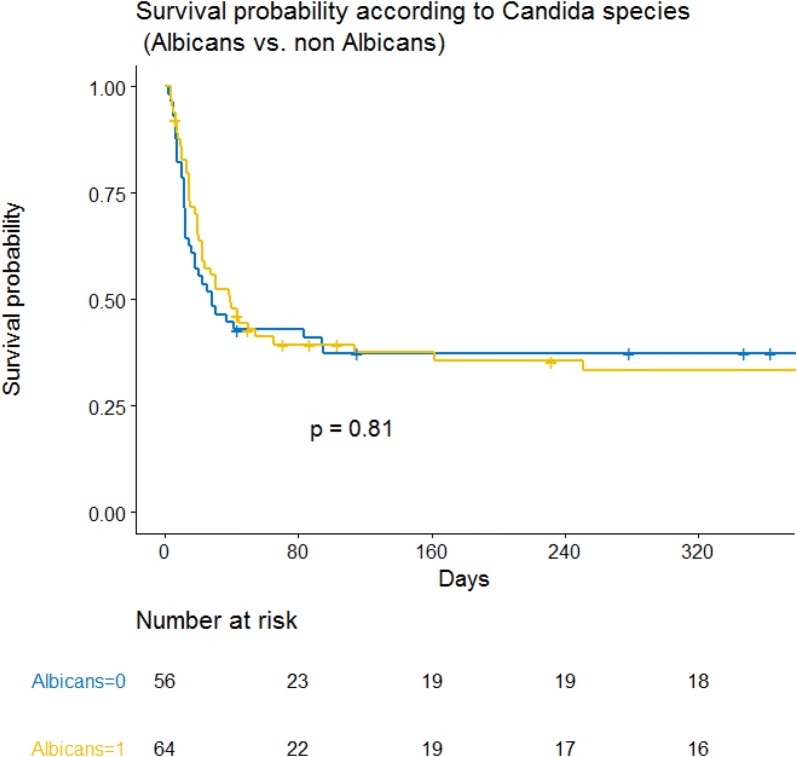
Kaplan–Meier survival curve. Survival probability according to *Candida* species (*albicans* vs non-*albicans*)

After adjustment for confounders, allogenic stem cell transplantation, vasopressors and invasive mechanical ventilation were independently associated with hospital mortality. *Candida* species were not significantly associated with outcome when forced in the final model and did not change this later (Table 2, Figs. 3, 4). Last, when forced in the Cox model, neither treatment strategies (echinocandin as first-line therapy) nor ICU admission years per quartile were selected or modified the model.

**Table 2:** Mortality associated factors (multivariate analysis. Cox model)

Mortality associated factors	HR	CI95%	*p* value
*Candida albicans*	0.98	0.60–1.6	0.93
Invasive mechanical ventilation	1.98	1.03–3.81	0.04
Vasopressors	1.85	1.12–3.07	0.02
Allogenic SCT	2.51	1.05–5.99	0.04

*SCT* stem cell transplantation, *HR* hazard ratio, *CI95%* confidence interval 95%

### Outcome in patients with and without candidemia

In a way to further explore the influence of candidemia on outcome, our study population was compared to a control group without candidemia and described elsewhere [16]. Main characteristics of patients with and without candidemia are reported in Additional file 1: Table S3. Distribution of propensity score and patients characteristic before and after matching, and changes in mean standardized difference and patients’ characteristics after matching are reported in Additional file 1: Figure S2, Figure S3, Figure S4 and Table S4. No influence of candidemia on mortality was noted before matching (Additional file 1: Figure S5), after matching (Additional file 1: Figure S6) or after adjustment for SOFA score at ICU admission (HR 0.78, 95%CI 0.58–1.09).

## Discussion

This study is the first to describe clinical features and outcome of this specific population. Hospital mortality in this setting was 60%. Patients’ severity as assessed by organ support and allogenic stem cell transplantation were independently associated with hospital mortality. Conversely, *Candida* species, susceptibility to antifungal therapies or neutropenia, had no influence on outcome.

The observed poor prognosis is concordant with previous studies in this field. Hence, overall mortality after candidemia has been reported to be up to 40% in the general population [1–4], rising to 50% in critically ill patients [1, 4] and 70% in patients with septic shock [7]. Mortality of onco-hematological patients with candidemia was reported to be of 40% [4, 17, 18]. Recently, Lortholary et al. [4] reported in a large prospective cohort of candidemia, a mortality of 50% for onco-hematological patients admitted in ICU with candidemia. Our data are in line with these reports, suggesting a high mortality associated with both underlying immune defect and underlying comorbidities.

Interestingly, in this study, most of the variables associated with outcome were surrogate of patients’ severity. These data are concordant with previous studies that reported higher mortality of critically ill patients with candidemia [1, 4]. In this line, Kollef et al. [7], reported a 69% mortality in patients with septic shock and candidemia. Although neutropenia has been associated with poor outcome in the general population of patients with candidemia [8], we were unable to detect such an effect in this study. The only exception is the peculiar population of allogeneic stem cell transplant recipients. These data are concordant with the poor prognosis of critically ill allogeneic stem cell transplant recipients, in whom a mortality of 51% was described, rising to 71% when mechanical ventilation was needed [19]. In this cohort, allogeneic stem cell transplant recipients with candidemia had a hospital mortality of 88%. This high mortality may reflect cumulative impact of mortality risk in this subgroup, direct influence of candidemia and the fact that candidemia may be a surrogate marker of severity or underlying immune defect in these patients [20, 21]. This later is further underlined by the lack of influence of candidemia on outcome after adjustment for confounders and when compared to a control group of patients without candidemia [16].

Last, several findings specific to candidemia and its management are to be noted. First, *Candida* specie and susceptibility had no influence on outcome. Some of the previous studies reported *Candida* species, namely *C. glabrata* or *C. parapsilosis* to be associated with better prognosis [4, 8, 22]. This association is, however, inconstantly reported in the literature [4, 5], and our results do not support such association. Our study may, however, lack statistical power to detect such an effect. Similarly, we were unable to detect the influence of management strategies on outcome. Neither initial therapy nor catheter withdrawal was associated with outcome while having been demonstrated factors associated with survival for patients with candidemia by previous studies [7, 8, 23], and being recommended by guidelines [6]. This may be explained by the homogenous management strategies in this study leading to high rate of catheter removal and systemic initiation of antifungal treatment the day of *Candida* identification. Nonetheless, the delay from onset of symptoms to antifungal therapy or catheter removal remained unacceptably long with a median of 3 days, reflecting time to culture positivity. Hence, despite rate of antifungal prophylaxis in this population (18%) and preemptive therapies, antifungal therapy remains dictated in this setting by culture positivity. This may reflect either a lack of clinical vignette specific enough to lead to adequate preemptive therapy initiation or failure to identify these vignettes. Development and validation of strategy that may allow reduction of this delay may be required. In this line, extension and validation of preemptive strategy in immunocompromised patients, excluded from recent trials [9, 10], validation of biomarkers driven strategies in this setting might deserve to be evaluated.

Our study has several limitations. First, the observational design and lack of a control group preclude any causality inference in this setting. In addition, this study was performed in only three centers, with high volume and experience of immunocompromised patients. This could explain the homogeneity in terms of patients cares and might constitute a selection bias. In this line, rate of hematological malignancies was high, and over-represented, in line with case mix in participating centers. Whether this may partly explain the lack of influence of underlying disease may deserve to be assessed by additional studies. In this line, the study period extends over a decade and changes in practices may have influenced our findings. We described two different groups of patients with candidemia, namely patients with ICU acquired candidemia and patient admitted in ICU for candidemia. If mixing these two groups might be misleading, we demonstrated similar features and outcomes of them. Moreover, despite the relatively large sample size in line with an uncommon disease, our negative findings might be related to the lack of statistical power. Thus, the lack of influence of *Candida* specie or *Candida* susceptibility and the absence of influence of management strategy may reflect the lack of statistical power rather than the lack of influence. Last, although we failed to observe an association between candidemia and mortality, this post hoc analysis was limited by selection bias in the control group. Since comparability across groups cannot be ensured after adjustment, these results are to be interpreted cautiously. Nonetheless, this post hoc analysis, although hypothesis generating, may suggest a lack of increased mortality in patients with candidemia after adjustment of case mix and patients’ severity that may deserve to be explored in future studies.

## Conclusion

Our results confirm the high mortality of candidemia in immunocompromised critically ill patients despite adequate first-line therapy and high adherence to recommendation in most patients. In this setting, initial severity and underlying allogenic stem cell transplantation are main factors associated with outcome, while *Candida* specie and susceptibility do not appear to be associated with outcome. Last, despite antifungal prophylaxis and use of preemptive antifungal therapy in neutropenic patients, antifungal therapy proves to be initiated 3 days after onset of candidemia, suggesting need for better risk stratification and validation of dedicated preemptive strategies.

## Additional file

**Additional file 1: Figure S1.** Venn diagram representing interaction between candida risk factors.** Figure S2.** Distribution of propensity score of having candidemia, in the study population ("treatment") and the control group ("control") (17), before and after matching.** Figure S3.** Distribution of propensity score of having candidemia, in the study population ("treatment") and the control group ("control") (17), before and after matching.** Figure S4.** Standardized mean difference across groups for accounted variables before and after matching. SOFA: Sepsis-related Organ Failure Assessment; RRT: Renal Replacement Therapy; MV: Mechanical ventilation; HSCT: Hematopoietic Stem Cell Transplantation.** Figure S5.** Kaplan-Meier Survival Curve in patients with Candidemia and in patients without candidemia, before matching (Difference tested using Log-Rank test).** Figure S6.** Kaplan-Meier Survival Curve in patients with Candidemia and in patients without candidemia, after matching (Difference tested using Log-Rank test).** Table S1.** Patients characteristics.** Table S2.** Comparison between ICU acquired candidemia and "primary candidemia" (Patients with candidemia developing before 24h of ICU admission).** Table S3.** Candidemia patients characteristics and control patients [16] before adjustment.** Table S4.** Candidemia Patients characteristics and control patients after propensity score matching on gender, organ support, underlying immune defect and stem cell transplantation.

## Data Availability

The datasets used and/or analysed during the current study are available from the corresponding author on reasonable request.

## Abbreviations

CI: confidence interval
ICU: intensive care unit
IQR: interquartile range
OR: odds ratio
SOFA: sepsis-related organ failure assessment
EUCAST: European Committee on Antimicrobial Susceptibility Testing
SCT: stem cell transplantation
