# Supplementary material for: Candidemia in critically ill immunocompromised patients: report of a retrospective multicenter cohort study
Source: Ann Intensive Care. 2019 Jun 3;9:62. doi: 10.1186/s13613-019-0539-2 (PMC6546779; doi:10.1186/s13613-019-0539-2)
Supplement: Supplementary file 1 — Additional file 1: Figure S1. Venn diagram representing interaction between candida risk factors. Figure S2. Distribution of propensity score of having candidemia, in the study population ("treatment") and the control group ("control") (17), before and after matching. Figure S3. Distribution of propensity score of having candidemia, in the study population ("treatment") and the control group ("control") (17), before and after matching. Figure S4. Standardized mean difference across groups for accounted variables before and after matching. SOFA: Sepsis-related Organ Failure Assessment; RRT: Renal Replacement Therapy; MV: Mechanical ventilation; HSCT: Hematopoietic Stem Cell Transplantation. Figure S5. Kaplan-Meier Survival Curve in patients with Candidemia and in patients without candidemia, before matching (Difference tested using Log-Rank test). Figure S6. Kaplan-Meier Survival Curve in patients with Candidemia and in patients without candidemia, after matching (Difference tested using Log-Rank test). Table S1. Patients characteristics. Table S2. Comparison between ICU acquired candidemia and "primary candidemia" (Patients with candidemia developing before 24h of ICU admission). Table S3. Candidemia patients characteristics and control patients [16] before adjustment. Table S4. Candidemia Patients characteristics and control patients after propensity score matching on gender, organ support, underlying immune defect and stem cell transplantation. [file 13613_2019_539_MOESM1_ESM.docx]

**Figure S1: Venn diagram representing interaction between candida risk factors.**

Colonization: Candida known colonization; RRT: Renal replacement therapy; Nutrition: ongoing parenteral nutrition
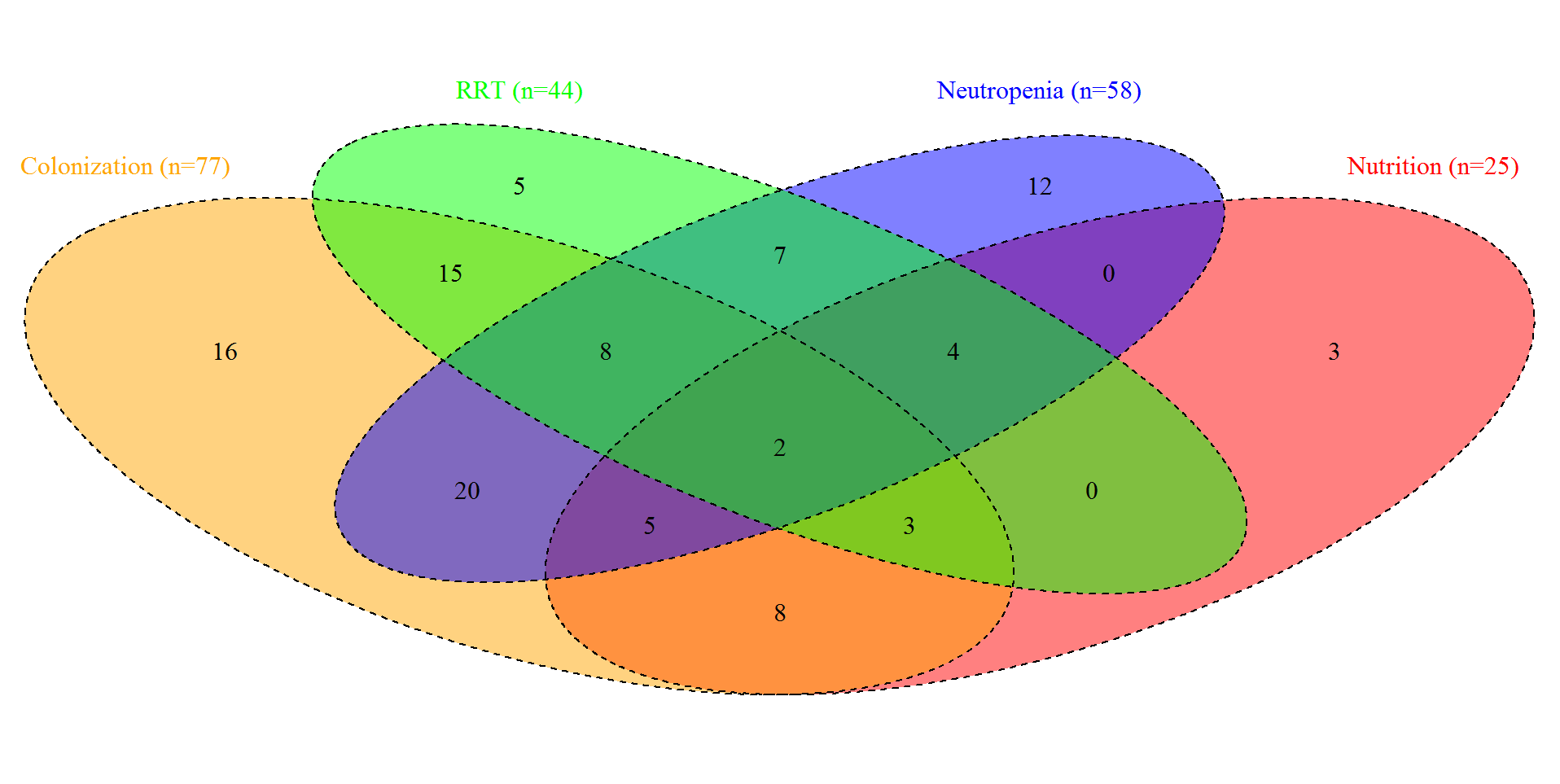


**Figure S2.** Distribution of propensity score of having candidemia, in the study population (“treatment”) and the control group (“control”) (manuscript reference #16), before and after matching.


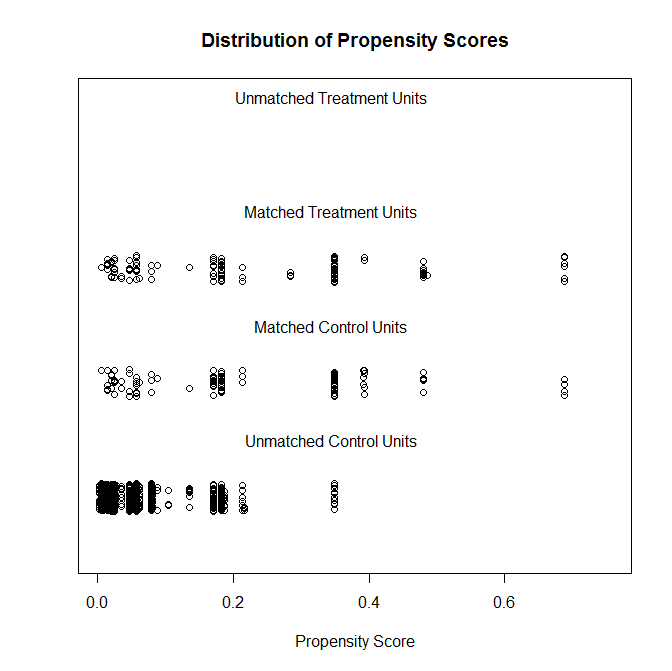
**Figure S3.** Distribution of propensity score of having candidemia, in the study population (“treatment”) and the control group (“control”) (manuscript reference #16), before and after matching.


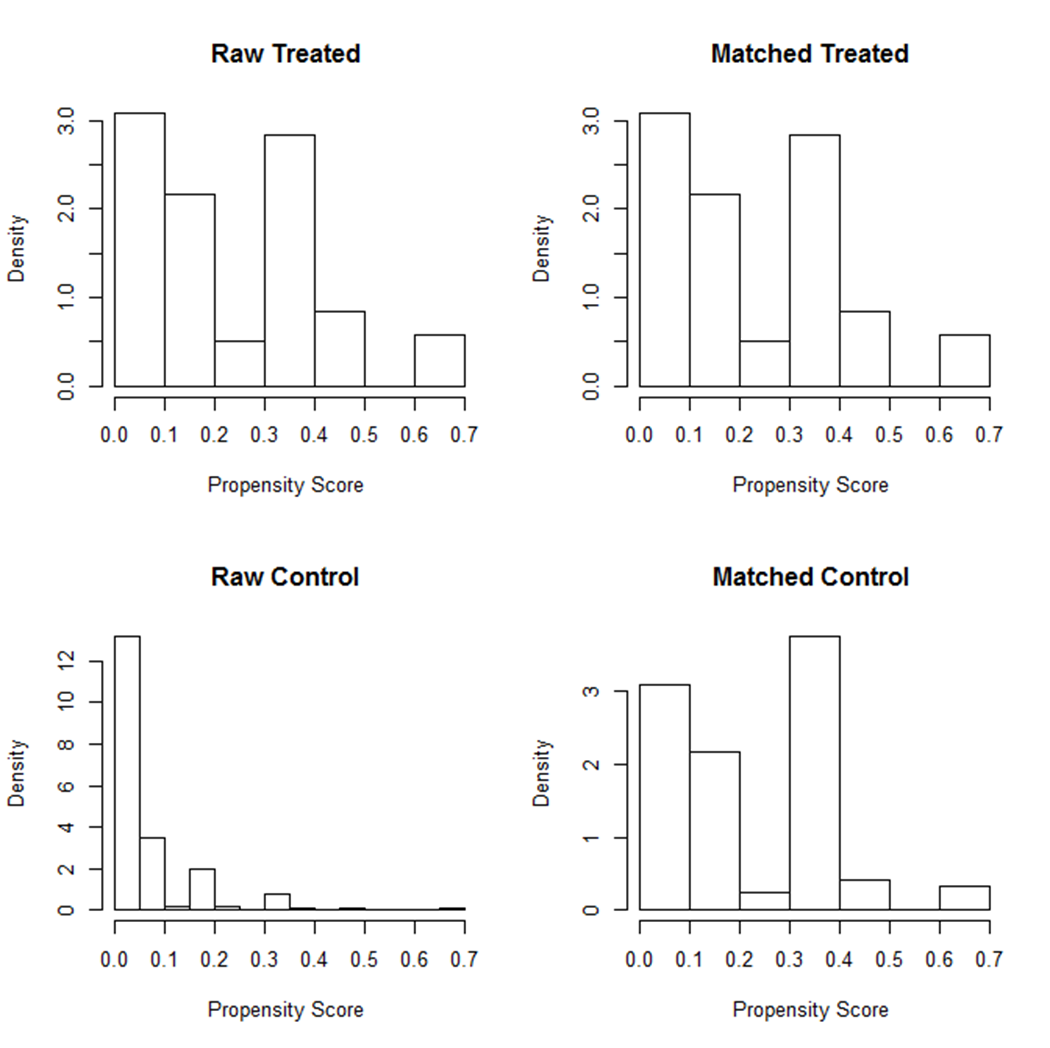


**Figure S4. Standardized mean difference across groups for accounted variables before and after matching. SOFA: Sepsis-related Organ Failure Assessment; RRT: Renal Replacement Therapy; MV: Mechanical ventilation; HSCT: Hematopoietic Stem Cell Transplantation.**


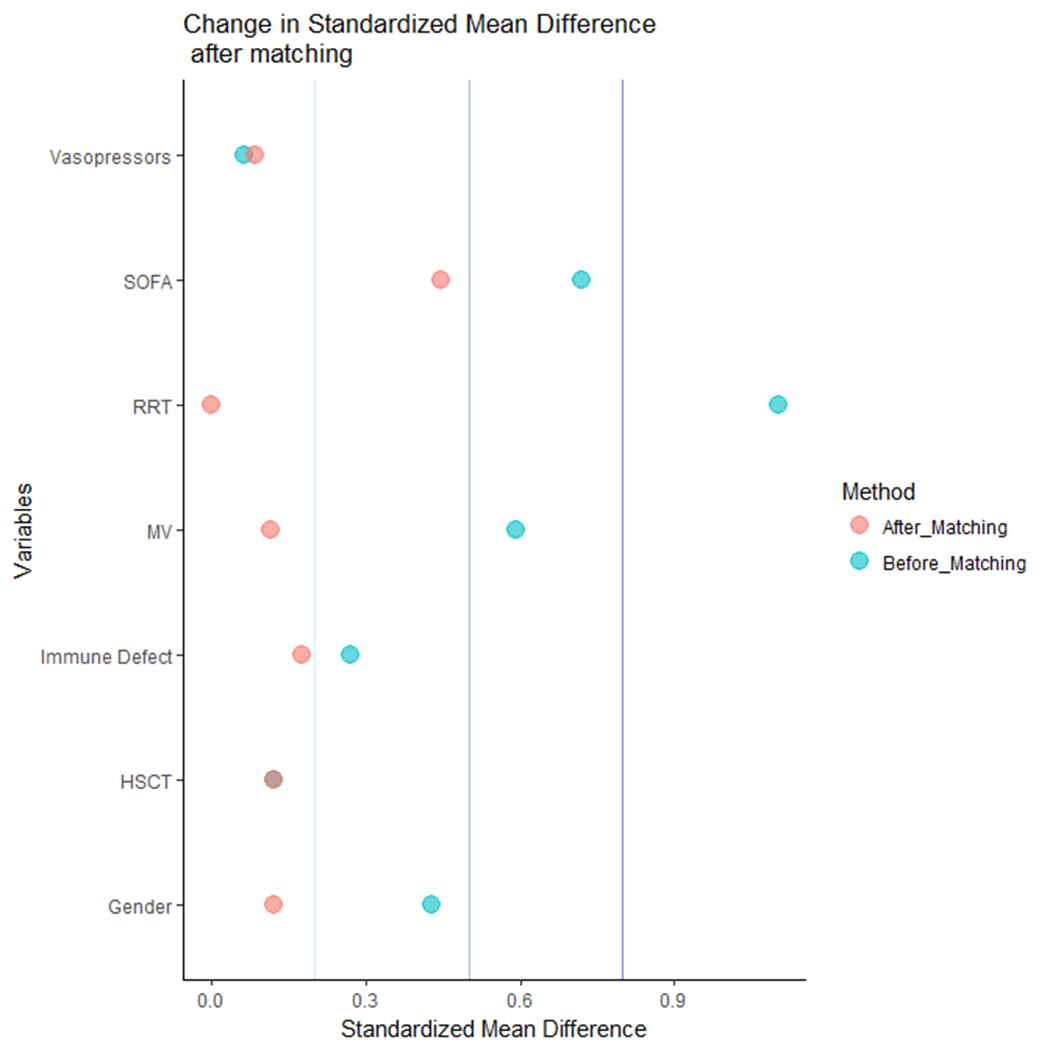


**Figure S5. Kaplan-Meier Survival Curve in patients with Candidemia and in patients without candidemia, before matching (Difference tested using Log-Rank test).**

**
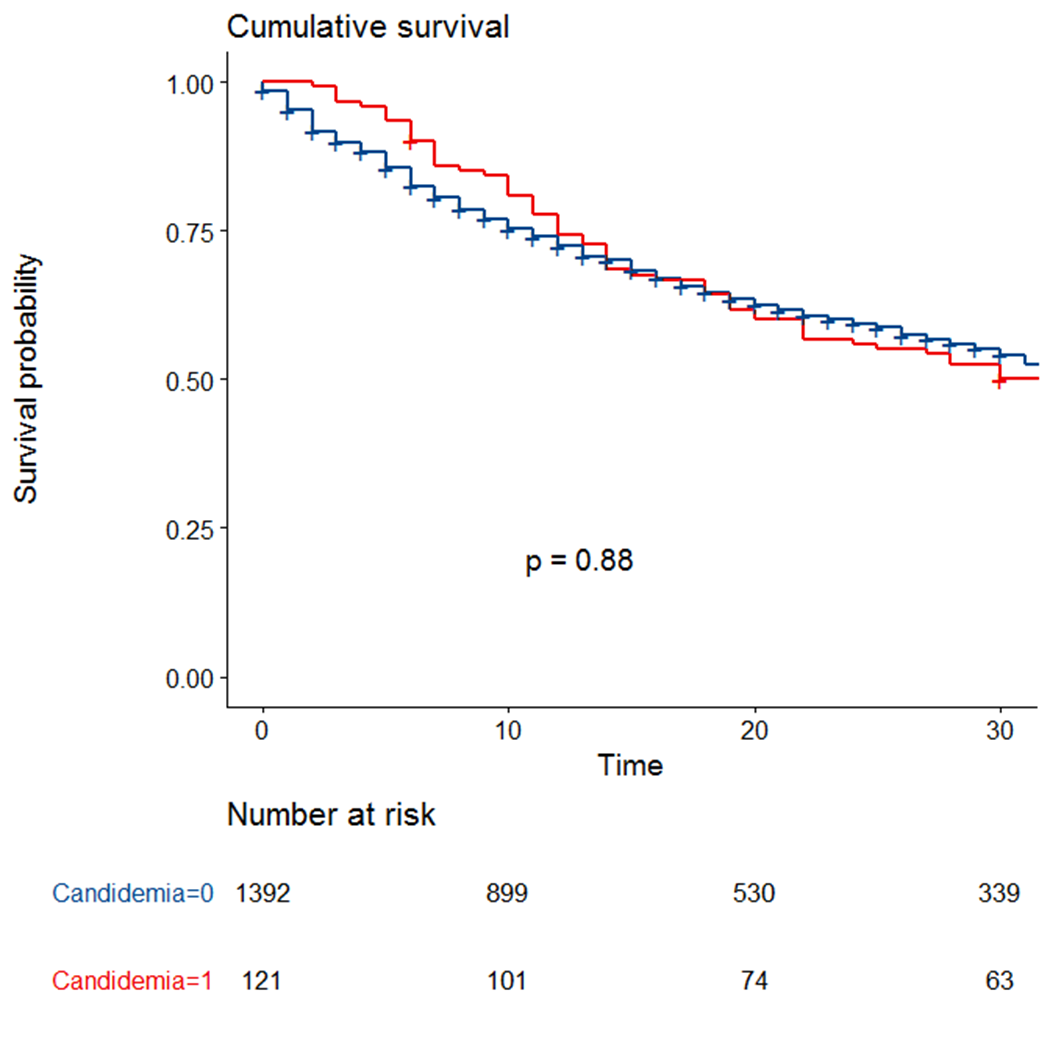
**

**Figure S6. Kaplan-Meier Survival Curve in patients with Candidemia and in patients without candidemia, after matching (Difference tested using Log-Rank test)**

**
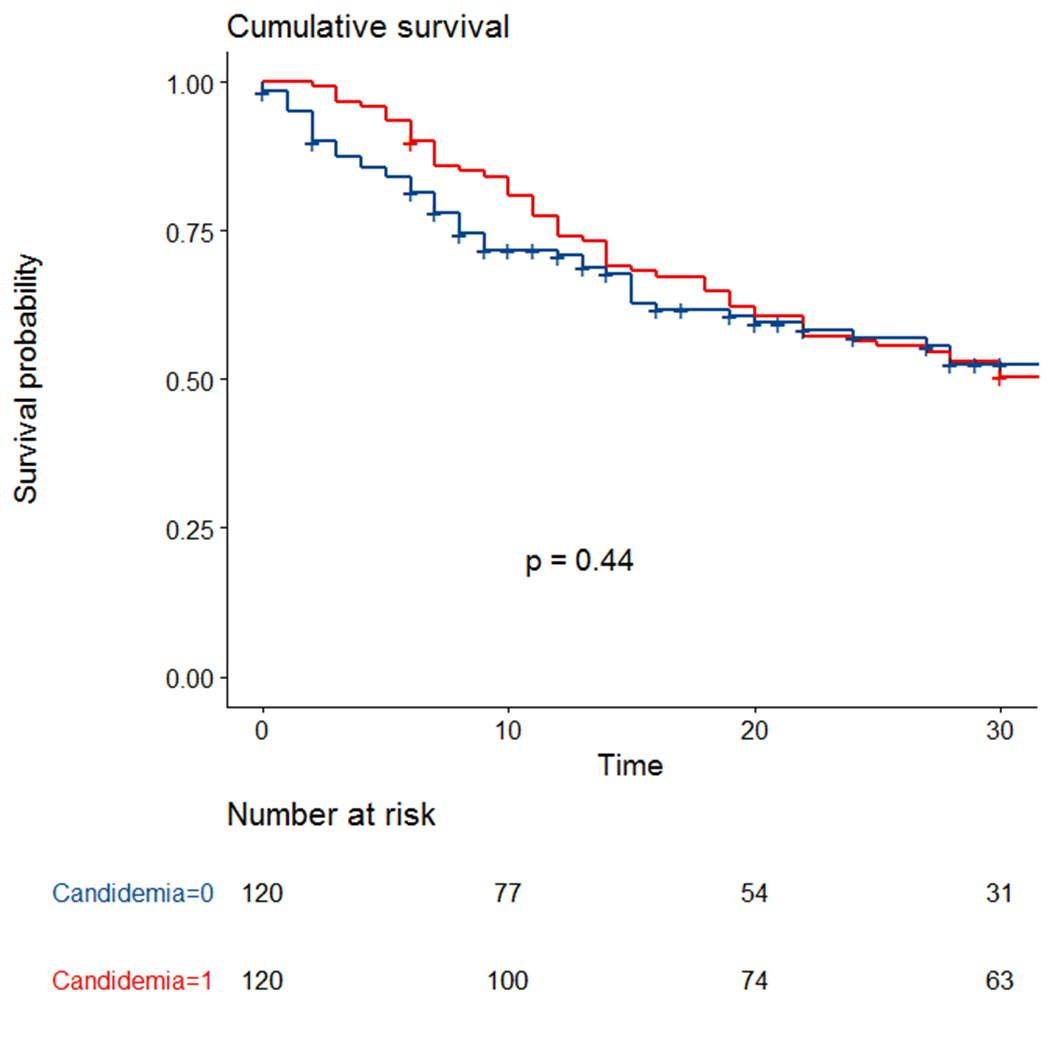
**

**Table S1: Patients characteristics**

| **Clinical features** | **Total**  **N= 121 (%)** |
| --- | --- |
| Female gender | 47 (39%) |
| Median age (IQR) | 60yo (49-66) |
| **Immunosuppression** |  |
| Malignant hemopathy | 81 (67%) |
| *Acute myeloid leukemia* | 17 (14%) |
| *Myelodysplasia* | 6 (5%) |
| *Acute lymphoid leukemia* | 9 (7%) |
| *Myeloma* | 8 (7%) |
| *Lymphoma* | 45 (37%) |
| *Autogenic SCT* | 12 (10%) |
| *Allogenic SCT* | 8 (7%) |
| Cancer | 36 (30%) |
| Autoimmune disease | 13 (11%) |
| Median time (IQR) between first immunosuppression and ICU admission | 193 days (29-832) |
| Median time (IQR) between last immunosuppression and ICU admission | 62 days (18-370) |
| **Candidemia risk factors** |  |
| Proton pump inhibitor | 48 (73%) |
| Antifungal prophylaxis | 20 (16.5%) |
| Past history of candidemia | 4 (3%) |
| Candida colonization | 77 (81%) |
| Presence of catheter | 103 (94%) |
| *Central venous catheter* | 103 (94%) |
| *Arterial catheter* | 51 (49%) |
| Parenteral nutrition | 25 (31%) |
| Recent (1 week) renal replacement therapy | 44 (38%) |
| Recent (2weeks) abdominal surgery | 14 (12%) |
| Neutropenia < 500/mm3 | 58 (49%) |
| HIV infection | 19 (16%) |
| Hypogammaglobulinemia | 12 (25%) |
| **ICU features** |  |
| Median (IQR) SOFA day 1 | 10 (6-15) |
| Vasopressors day 1 | 66 (54,5%) |
| Vasopressors during hospitalization | 91 (75%) |
| Invasive mechanical ventilation during hospitalization | 91 (75%) |
| Renal replacement therapy during hospitalization | 71 (61%) |
| Surgery | 25 (21%) |
| **Candidemia features** |  |
| ICU admission with candidemia | 51 (42%) |
| ICU acquired candidemia | 70 (58%) |
| Candida specie |  |
| *Albicans* | 65 (54%) |
| *Glabrata* | 23 (19%) |
| *Tropicalis* | 13 (11%) |
| *Parapsilosis* | 9 (7%) |
| *Krusei* | 9 (7%) |
| *Dubliniensis* | 5 (4%) |
| *Lusitaniae* | 4 (3%) |
| *Kefyr* | 2 (2%) |
| *Utilis* | 1 (1%) |
| *Inconspicua* | 1 (1%) |
| Median time to blood culture positivity (range) | 3 days (1-6) |
| Median time to first negative blood culture (range) | 1 day (1-25) |
| Fluconazole susceptibility | 61 (70%) |
| Echinocandins susceptibility | 73 (92%) |
| Localizations |  |
| *Enterocolitis* | 51 (55%) |
| *Positive urine culture* | 35 (47%) |
| *Endophthalmitis* | 4 (10%) |
| *Cutaneous secondary lesion* | 10 (8%) |
| *Thrombosis* | 10 (8%) |
| *Peritoneal infection* | 5 (4%) |
| *Lung abscess / pleural infection* | 3 (3%) |
| *Kidney abscess* | 3 (3%) |
| *Cerebral abscess* | 2 (2%) |
| *Hepatosplenic candidiasis* | 2 (2%) |
| *Endocarditis* | 0 (0%) |
| *Osteo-articular infection* | 0 (0%) |
| **Treatment of candidemia** |  |
| Antifungal treatment | 119 (98%) |
| Median time from candidemia to antifungal treatment (range) | 3 days (1-5) |
| First line |  |
| *Echinocandin* | 69 (57%) |
| *Fluconazole* | 27 (22%) |
| *Lipid formulation Amphotericin B* | 13 (10%) |
| *Amphotericin B Deoxycholate* | 8 (7%) |
| *Voriconazole* | 2 (2%) |
| *Appropriate first line therapy* | 78 (90%) |
| Fluconazole de-escalation | 39 (41%) |
| Catheter removal | 103 (97%) |
| Median time from candidemia to catheter removal (range) | 3 days (1-17) |

SCT: Stem Cell Transplantation; ICU: Intensive Care Unit; IQR: Interquartile Range.

**Table S2. Comparison between ICU acquired candidemia and “primary candidemia” (Patients with candidemia developing before 24h of ICU admission)**

| **Clinical features** | **ICU acquired**  **N = 70** | **Primary**  **N = 51** | ***p* value** |
| --- | --- | --- | --- |
| Female gender | 32 (46%) | 15 (29%) | 0.07 |
| Age | 60 (49-65) | 61 (49-67) | 0.73 |
| Median Year of ICU admission (IQR) | 2013 (2010-2016) | 2013 (2010-2015) | 0.75 |
| **Underlying immunosuppression** |  |  |  |
| Solid tumor | 20 (29%) | 15 (29%) | 0.91 |
| Hematological malignancy | 44 (63%) | 37 (72%) | 0.26 |
| *Allogenic SCT* | 3 (4%) | 5 (10%) | 0.28 |
| *Acute myeloid leukemia* | 10 (14%) | 7 (14%) | 0.93 |
| *Acute lymphoid leukemia* | 5 (7%) | 4 (8%) | 1.00 |
| *Lymphoma* | 22 (31%) | 23 (45%) | 0.12 |
| *Myelodysplasia* | 1 (1.4%) | 5 (10%) | 0.08 |
| Autoimmune disease | 12 (17%) | 1 (2%) | 0.007 |
| HIV infection | 12 (18%) | 7 (14%) | 0.61 |
| Neutropenia | 29 (43%) | 29 (57%) | 0.09 |
|  |  |  |  |
| **ICU features** |  |  |  |
| SOFA score | 10.5 (7-15) | 11 (6-17) | 0.81 |
| Renal replacement therapy | 48 (71%) | 24 (47%) | 0.02 |
| Vasopressors | 42 (60%) | 24 (47%) | 0.15 |
| **Candidemia features** |  |  |  |
| *Candida albicans* | 40 (57%) | 25 (49%) | 0.38 |
| Fluconazole susceptibility | 35 (73%) | 27 (67%) | 0.58 |
| Adequacy of first AF therapy | 42 (86%) | 36 (95%) | 0.29 |
| **Outcomes** |  |  |  |
| ICU survival | 30 (43%) | 28 (56%) | 0.19 |
| Hospital survival | 21 (32%) | 20 (41%) | 0.29 |

SCT: Stem Cell Transplantation; ICU: Intensive Care Unit; IQR: Interquartile Range; AF: Antifungal.

**Table S3: Candidemia patients’ characteristics and control patients (manuscript reference #16) before adjustment**

|  | No candidemia  N=1392 | Candidemia  N=121 | p |
| --- | --- | --- | --- |
| Female Gender | 830 (60%) | 47 (39%) | <0.001 |
| Underlying immune defect | | | 0.023 |
| Hematological malignancy | 770 (55%) | 81 (67%) |  |
| Solid tumor | 434 (31%) | 32 (26%) |  |
| Auto-immune disease | 188 (14%) | 8 (7%) |  |
| Allogeneic SCT | 138 (10%) | 8 (7%) | 0.308 |
| SOFA Score | 7 [4-10] | 11 [6-15] | <0.001 |
| Vasopressors | 798 (57%) | 66 (55%) | 0.691 |
| Renal Replacement Therapy | 214 (15%) | 76 (63%) | <0.001 |
| Mechanical Ventilation | 686 (49%) | 92 (77%) | <0.001 |
| Hospital mortality | 613 (44%) | 74 (61%) | <0.001 |

**Table S4: Candidemia Patients characteristics and control patients after propensity score matching on gender, organ support, underlying immune defect and stem cell transplantation.**

|  | No candidemia  N=120 | Candidemia  N=120 | p |
| --- | --- | --- | --- |
| Female Gender | 40 (33%) | 47 (39%) | 0.420 |
| Underlying immune defect | | | 0.395 |
| Hematological malignancy | 78 (65%) | 81 (67%) |  |
| Solid tumor | 38 (32%) | 36 (30%) |  |
| Auto-immune disease | 4 (3%) | 12 (10%) |  |
| Allogeneic SCT | 12 (10%) | 8 (7%) | 0.484 |
| SOFA Score | 8 [5-11] | 10 [6-15] | 0.002 |
| Vasopressors | 70 (58%) | 65 (54%) | 0.60 |
| Renal Replacement Therapy | 75 (63%) | 71 (62%) | 0.98 |
| Mechanical Ventilation | 86 (72%) | 91 (75%) | 0.42 |
| Hospital mortality | 63 (53%) | 73 (61%) | 0.24 |
